# Differential Effects of Antiseptic Mouth Rinses on SARS-CoV-2 Infectivity In Vitro

**DOI:** 10.3390/pathogens10030272

**Published:** 2021-03-01

**Authors:** Chuan Xu, Annie Wang, Eileen R. Hoskin, Carla Cugini, Kenneth Markowitz, Theresa L. Chang, Daniel H. Fine

**Affiliations:** 1 Public Health Research Institute, Rutgers, New Jersey Medical School, 225 Warren Street, Newark, NJ 07103, USA; cx89@njms.rutgers.edu (C.X.); aw768@njms.rutgers.edu (A.W.); 2 Department of Restorative Dentistry, School of Dental Medicine, Rutgers, The State University of New Jersey, Newark, NJ 07103, USA; hoskiner@sdm.rutgers.edu; 3 Department of Oral Biology, School of Dental Medicine, Rutgers, The State University of New Jersey, Newark, NJ 07103, USA; cc1337@sdm.rutgers.edu (C.C.); markowkj@sdm.rutgers.edu (K.M.); 4 Department of Microbiology, Biochemistry and Molecular Genetics, New Jersey Medical School, Rutgers, The State University of New Jersey, Newark, NJ 07103, USA

**Keywords:** mouth rinses, antiseptics, SARS-CoV-2

## Abstract

Severe acute respiratory syndrome-related coronavirus (SARS-CoV-2) is detectable in saliva from asymptomatic individuals, suggesting a potential benefit from the use of mouth rinses to suppress viral load and reduce virus spread. Published studies on the reduction of SARS-CoV-2-induced cytotoxic effects by mouth rinses do not exclude antiseptic mouth rinse-associated cytotoxicity. Here, we determined the effect of commercially available mouth rinses and antiseptic povidone-iodine on the infectivity of replication-competent SARS-CoV-2 viruses and of pseudotyped SARS-CoV-2 viruses. We first determined the effect of mouth rinses on cell viability to ensure that antiviral activity was not a consequence of mouth rinse-induced cytotoxicity. Colgate Peroxyl (hydrogen peroxide) exhibited the most cytotoxicity, followed by povidone-iodine, chlorhexidine gluconate (CHG), and Listerine (essential oils and alcohol). The potent antiviral activities of Colgate Peroxyl mouth rinse and povidone-iodine were the consequence of rinse-mediated cellular damage when the products were present during infection. The potency of CHG was greater when the product was not washed off after virus attachment, suggesting that the prolonged effect of mouth rinses on cells impacts the antiviral outcome. To minimalize mouth rinse-associated cytotoxicity, mouth rinse was largely removed from treated viruses by centrifugation prior to infection of cells. A 5% (*v*/*v*) dilution of Colgate Peroxyl or povidone-iodine completely blocked viral infectivity. A similar 5% (*v*/*v*) dilution of Listerine or CHG had a moderate suppressive effect on the virus, but a 50% (*v*/*v*) dilution of Listerine or CHG blocked viral infectivity completely. Mouth rinses inactivated the virus without prolonged incubation. The new infectivity assay, with limited impacts of mouth rinse-associated cytotoxicity, showed the differential effects of mouth rinses on SARS-CoV-2 infection. Our results indicate that mouth rinses can significantly reduce virus infectivity, suggesting a potential benefit for reducing SARS-CoV-2 spread.

## 1. Introduction

Severe acute respiratory syndrome-related coronavirus (SARS-CoV-2), a non-segmented positive-strand RNA enveloped virus, is the causative agent of coronavirus disease 19 (COVID-19). As of February 2021, COVID-19 spread has resulted in more than 111 million cases worldwide and more than 28 million cases in the United States alone [1,2]. SARS-CoV-2 particles have a spherical shape with diameters ranging from 60–140 nm and are coated with 9–12 nm spike proteins together with the envelope and membrane proteins, which are embed in a lipid-bilayer viral envelope [3]. Inside the envelope, the single-stranded positive-sense viral RNA is associated with nucleocapsid proteins [4,5]. Targeting viral components, including the membrane (envelope), surface spike proteins, and nucleic acids, to inactive viruses may reduce transmission. Evidence indicates that transmission of SARS-CoV-2 occurs through virus-containing secretions, such as saliva and respiratory secretions, or their droplets [6]. Salivary SARS-CoV-2 viral load is highest during the first week after symptom onset [7], and individuals with SARS-CoV-2 infection shed virus and can remain asymptomatic for a prolonged period [8,9], highlighting the importance of developing a strategy to prevent virus spread in the general population. Additionally, there is an urgent need for evidence-based practices to protect patients and healthcare workers in the dental office and elsewhere when salivary droplets and aerosols are generated during dental treatment when masks for patients are not an option.

Antiseptic mouth rinses have been shown to have efficacy in reducing bacteria and viruses in the oral cavity and in dental aerosols [10,11,12]. The antiseptic Listerine and chlorhexidine gluconate 0.12% (CHG) have been shown to reduce herpes simplex virus-1 load in saliva after rinsing [13,14]. Potential inhibitory effects of mouth rinses on SARS-CoV-2 inactivation have been proposed based on the assumption that the organic components in the mouth rinses disrupt viral membranes or act on viral proteins [15,16,17]. Because viable cells are required for productive infection, toxic effects on cells that may produce unfavorable conditions for viral infection can be misinterpreted as a potent antiviral activity. While most commercially available mouth rinses are safe, particularly after dilution in the oral cavity, their effects on the infectivity of the virus may be overestimated if mouth rinse-associated cytotoxicity is not considered. Recent studies demonstrated the effect of mouth rinses and povidone-iodine, a common topical antiseptic, on SARS-CoV-2 infection, but the studies did not discriminate between the effect of these products on viral infectivity and their cytotoxic effect on epithelial cells [18,19,20]. For example, in the study by Bidra et al., a mixture of SARS-CoV-2 viruses and diluted povidone-iodine was added to Vero cells for 5 days, followed by determination of the cytopathic effects. The same assay was conducted by Meister et al., wherein cell viability was determined by crystal violet staining, a method that does not directly distinguish live and dead cells. Antiseptic-associated cell death can result in decreased numbers of target cells for viral infection, producing an apparent decrease in viral infectivity, which can be interpreted as a potent antiviral effect.

Here, we determined the effect of mouth rinses, including Listerine, chlorhexidine gluconate (CHG), and Colgate Peroxyl, on cell viability prior to assessing their impact on the infectivity of SARS-CoV-2 viruses. We included povidone-iodine as a comparison because a published study suggested its use as a mouth rinse [18]. We used replication-competent SARS-CoV-2 viruses expressing mNeonGreen, which allowed us to monitor the green signal in live cells within 24 h after infection, which avoided significant virus-induced cytopathic effects seen at later time points. Importantly, we used a cell imaging multi-mode reader to monitor viral infection (fluorescent signals) and cell morphology simultaneously. We also used a single-cycle infection assay using a pseudotyped SARS-CoV-2 virus expressing SARS-CoV-2 spike proteins, which provides a non-pathogenic vector for assessing viral infectivity. The pseudotyped virus does not cause virus-induced cytotoxic effects but allows us to assess SARS-CoV-2 spike protein-mediated viral entry. Additionally, the assay would be useful for researchers without access to a biosafety Level 3 (BSL3) facility. We tested the effects of serial dilutions of the mouth rinses to determine their relative effectiveness against the virus directly as opposed to their cytotoxicity against mammalian cells.

## 2. Materials and Methods

### 2.1. Reagents

The infectious-clone-derived SARS-CoV-2 virus (USA_WA1/2020 strain) expressing mNeonGreen was kindly provided by Pei-Yong Shi at the University of Texas Medical Branch, Galveston, TX, USA [21]. A recombinant construct used for infectivity assays (pseudotyped SARS-CoV-2) was derived from the full-length SARS-CoV-2-Wuhan-Hu-1 surface (spike) gene (GenBank accession number QHD43416) [22], which was codon optimized for humans and synthesized with Kozak-START GCCACC ATG and STOP codons, flanked by 5′ Nhel/3′Apal sites for subcloning into the pcDNA3.1(+) vector (Thermo Fisher Scientific, USA). HEK293T cells and Vero E6 cells were purchased from American Type Culture Collection (ATCC), Manassas, VA, USA. Monoclonal antibody (Ab) against SARS-CoV-2 spike protein (IgG1 clone#43, Cat # 40591-MM43) was purchased from Sino Biological, Inc. (Wanye, PA, USA). Listerine Original (Johnson & Johnson Consumer Inc., Skillman, NJ, USA), povidone-iodine 10% (1% available iodine, CVS Pharmacy Inc., Woonsocket, RI, USA), Colgate Peroxyl (1.5% *w*/*v* hydrogen peroxide, Colgate-Palmolive Inc., New York, NY, USA), and chlorhexidine gluconate 0.12% (Xttrium Laboratories Inc., Mount Prospect, IL, USA) (Table 1) were purchased from a local pharmacy.

### 2.2. Cell Culture

HEK293T cells and human angiotensin-converting enzyme 2 (hACE2)-expressing HeLa cells (kindly provided by Dennis Burton, The Scripps Research Institute, La Jolla, CA, USA) [23] were cultured in Dulbecco’s Modified Eagle’s Medium (DMEM) supplemented with 10% fetal bovine serum (FBS). Vero E6 cells were cultured in Eagle’s Essential Minimal Medium (EMEM) with 5% FBS. TR146 cells were cultured in Ham’s F12 medium with glutamate and 10% FBS. DMEM, EMEM, Ham’s F12, and FBS were purchased from Millipore-Sigma (St. Louis, MO, USA). The choices of culture media were based on ATCC recommendations. Cell lines were maintained using standard tissue culture procedures.

### 2.3. Viral Infection

Infection assays are summarized in Supplementary Figure S1. Replication-competent SARS-CoV-2 viruses expressing mNeonGreen were propagated in Vero E6 cells as described previously [21]. All experiments were performed in a BSL3 laboratory. Powered air-purifying respirators (Breathe Easy, 3M), Tyvek suits, aprons, sleeves, booties, and double gloves were worn. Virus titers were determined by plaque assays. Briefly, Vero E6 cells were seeded at 6 × 10^5^ cells per well in a 6-well plate and cultured overnight. Cells were then exposed to serial dilutions of SARS-CoV-2 viruses for 1.5–2 h. After removing unbound viruses, cells were overlaid with 0.8% Agarose LE (Millipore-Sigma, St. Louis, MO, USA) in DMEM with 2% FBS. On post-infection Day 3, cells were fixed with 10% formaldehyde (in phosphate buffered saline (PBS) for 30 min. Agarose plugs were removed and fixed cells were stained with 0.2% crystal violet (*w*/*v*) in ethanol.

For the infection assay, Vero E6 cells at 1 × 10^4^ cells per well were incubated overnight in black 96-well glass bottom plates (Greiner Bio-One, Monroe, NC, USA). Cells were exposed to treated or untreated viruses in 50 μL at a multiplicity of infection (MOI) of 5 for 1 h followed by the addition of 100 μL FluoroBrite medium containing 2% FBS. The fluorescence signals from productive viral infection and cell images were monitored at 24 h after infection using a Biotek Cytation 5 (Winooski, VT, USA).

For single-cycle infection assays, replication-defective Human Immunodeficiency Virus (HIV)-1 luciferase-expressing reporter viruses pseudotyped with SARS-CoV-2 spike (S) proteins were produced by co-transfection of a plasmid encoding the envelope-deficient HIV-1 NL4-3 virus with the luciferase reporter gene (pNL4-3.Luc.R+ E-, kindly provided by Nathaniel Landau, New York University) and a pcDNA3.1 plasmid expressing the SARS-CoV-2 glycoprotein into HEK293T cells using Lipofectamine 3000 (Thermo Fisher Scientific, Waltham, MA, USA). The supernatant was collected 48 h after transfection and filtered. Virus stocks were analyzed for the HIV-1 p24 antigen by the AlphaLISA HIV p24 kit (PerkinElmer, Waltham, MA, USA) on a 2300 EnSpire Multilabel Plate Reader (PerkinElmer) according to the manufacturer’s instruction. Virus stocks contained approximately 200 ng/mL of HIV p24 protein.

For infection assays, cells were seeded at 5 × 10^4^ cells/well in a 48-well plate and cultured overnight. Pseudotyped SARS-CoV-2 luciferase reporter viruses were incubated with or without mouth rinse for 30 min at 37 °C before being added to HeLa-hACE2 cells. After 1–2 h viral attachment, infected cells were cultured in media with 10% FBS for 48–72 h. Cells were then lysed in a 1× passive lysis buffer (Promega Inc., Madison, WI, USA), followed by measuring luciferase activity (relative light units; RLUs) using Luciferase Substrate Buffer (Promega Inc.) on a 2300 EnSpire Multilabel Plate Reader.

To assess the effect of mouth rinses on the viruses, mouth rinse-treated SARS-CoV-2 viruses were concentrated by centrifugation at 14,000 rpm in a centrifuge (Eppendorf) at 4 °C for 2 h as described previously [24]. After removing the mouth rinses or medium (control samples), virus pellets were resuspended in DMEM and used to infect HeLa-hACE2 cells. Infection was determined by measuring fluorescence intensity after 24 h for replication-competent viruses or luciferase activity after 48 h for pseudotyped viruses.

### 2.4. Cytotoxicity Assay

HeLa-hACE2 and TR146 cells were plated in 96-well plates at 5000 cells per well and then treated with various dilutions of mouth rinses for the times indicated in the figure legends. Each treatment was performed in triplicate. Cell viability was analyzed using the CellTiter 96 AQueous One Solution Cell Proliferation Assay (Promega, Madison, WI, USA) according to the manufacture’s instruction. The signal was recorded as absorbance at 490 nm on a 96-well plate reader. The assay reagent contains a tetrazolium compound (3-(4,5-dimethylthiazol-2-yl)-5-(3-carboxymethoxyphenyl)-2-(4-sulfophenyl)-2H-tetrazolium, inner salt; MTS) and an electron coupling reagent (phenazine ethosulfate; PES) to measure metabolically active cells.

### 2.5. Statistical Analysis

Statistical comparisons were performed using one-way ANOVA with Dunnett’s multiple comparisons test. Prism 8 (GraphPad Software, LLC, Sand Diego, CA, USA) was used. *p* < 0.05 was considered significant.

## 3. Results

### 3.1. Differential Effects of Mouth Rinses on Cell Viability

**Figure 1:**
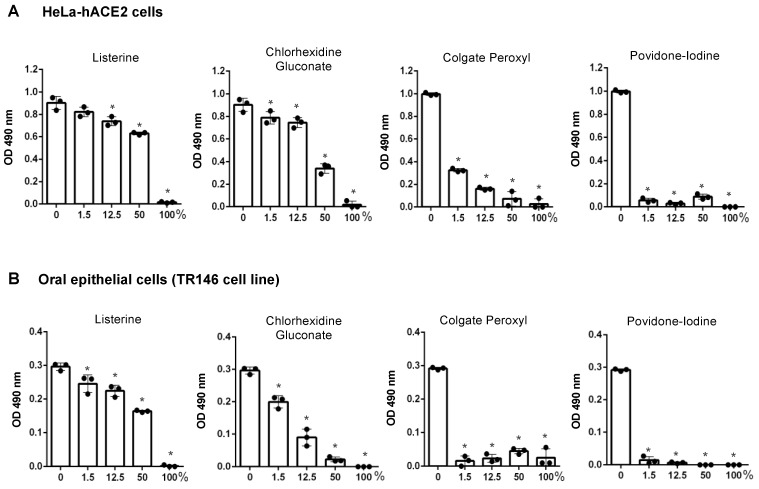
The effect of short-term exposure to mouth rinses on the viability of HeLa-hACE2 and oral epithelial cells. Human angiotensin-converting enzyme 2 (hACE2)-expressing HeLa cells (**A**) and oral epithelial TR146 cells (**B**) were treated for 20 s with different dilutions (*v*/*v*) of products including Listerine, chlorhexidine gluconate (CHG), Colgate Peroxyl, or povidone-iodine. Cells were washed and cultured with fresh medium immediately. Cell viability was determined by the 3-(4,5-dimethylthiazol-2-yl)-5-(3-carboxymethoxyphenyl)-2-(4-sulfophenyl)-2H-tetrazolium, inner salt (MTS)-based CellTiter 96 AQueous One Solution Cell Proliferation Assay. Data are means ±SD of three samples. The significance of differences between mouth rinse-treated cells and mocked-treated controls was compared; * *p* < 0.05.

It is critical to assess antiviral agents under non-cytotoxic conditions, as viruses depend on viable host cells for productive infection. Therefore, we first determined the effect of preincubation with mouth rinses on cell viability. Cells were exposed to mouth rinses for 20 s or for 2 h to simulate the immediate action of mouth rinses in the oral cavity and the in vitro viral infection assay, respectively. We then assessed the effect of mouth rinses on viruses with or without preincubation. Note that percentage dilutions (*v*/*v*) of commercial mouth rinse products are referenced in this study. For example, in Figure 1, 50% (*v*/*v*) CHG represents a solution composed of equal volumes of culture media and of the commercial product, and does not indicate the final concentration of active ingredients. HeLa-hACE2 cells were treated with serial dilutions in medium of Listerine, CHG, Colgate Peroxyl, or povidone-iodine for 20 s, washed, cultured in fresh media, and cell viability was determined. All undiluted mouth rinses (100% *v*/*v*) were highly toxic to HeLa-hACE2 and oral epithelial cells (Figure 1). Listerine was the least cytotoxic, followed closely by CHG. Both 1.5% (*v*/*v*) dilutions of Colgate Peroxyl and povidone-iodine were highly toxic to cells.

**Figure 2:**
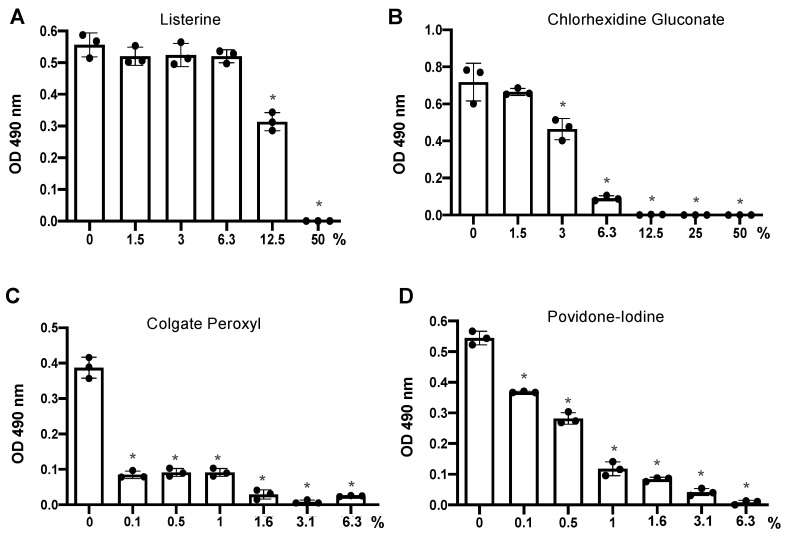
The effect of prolonged exposure to mouth rinses on cell viability. hACE2-expressing HeLa cells were treated for 2 h with serial dilutions of products including Listerine (**A**), CHG (**B**), Colgate Peroxyl (**C**), or povidone-iodine (**D**) starting at 50% (*v*/*v*), except povidone-iodine, which was started at 6.25% (*v*/*v*). Cell viability was determined by the MTS-based CellTiter 96 AQueous One Solution Cell Proliferation Assay. Data are means ±SD of three samples and are representative of two independent experiments. The significance of differences between mouth rinse-treated cells and mocked-treated controls was compared; * *p* < 0.05.

Infection requires 2 h viral attachment. We then determined the effect of 2 h exposure of mouth rinses on cell viability for comparison with the duration of viral attachment in the infection assay. We found that 6.3% (*v*/*v*) diluted Listerine and 1.5% (*v*/*v*) diluted CHG did not impact cell viability, whereas 0.1% (*v*/*v*) diluted Colgate Peroxyl or povidone-iodine significantly affected cell viability after 2 h exposure (Figure 2).

### 3.2. Antiviral Effect of Diluted Povidone-Iodine or Colgate Peroxl Was Associated with Cytotoxicity

**Figure 3:**
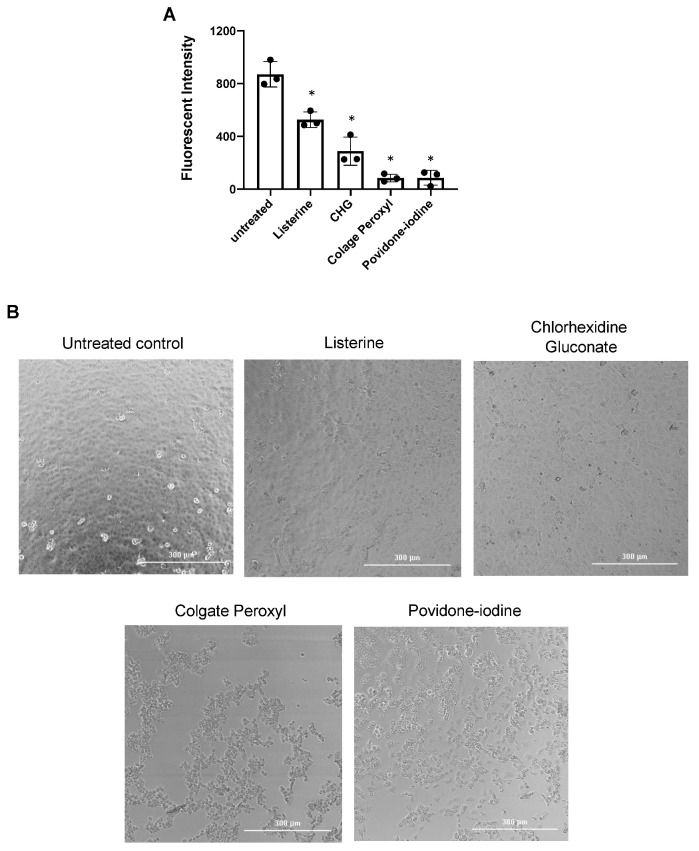
Effect of diluted antiseptics on infection by replication-competent severe acute respiratory syndrome-related coronavirus (SARS-CoV-2) virus when antiseptics were present in the culture. (**A**) Replication competent virus SARS-CoV-2 expressing mNeonGreen (multiplicity of infection (MOI) of 5) were mixed or not with Listerine (3%), CHG (1.5%), povidone-iodine (0.1%), or Colgate Peroxyl (0.05%), and immediately added (in 50 μL) to Vero cells and incubated 1 h for viral attachment. Antiseptics were diluted by the addition of 100 μL of the medium to reduce potential toxic effects. Fluorescence intensity derived from productive viral infection was determined at 24 h after infection. (**B**) Cell images were acquired on a Bioteck Cystatin 5 plate reader. Differences between mouth rinse-treated viruses and the medium control (0%) were compared; * *p* < 0.05. Data are means ±SD and are representative of three independent experiments.

Previous studies on the effect of antiseptics on SARS-CoV-2 infection used methods involving virus-induced cytopathic effects without excluding mouth rinse-associated cytotoxic effects [18,19,20]. To examine this more closely, we assessed the effect of highly diluted mouth rinses and povidone-iodine on replication-competent SARS-CoV-2 viruses without washing off the antiseptics; cell morphology was monitored as a crude measure of cytopathic effects. Viruses were treated with non-cytotoxic dilutions of Listerine and CHG, highly dilute Colgate Peroxyl, and low cytotoxic dilutions of povidone-iodine (Figure 2), and were immediately added to Vero cells. Additional media were added 2 h after infection, and cells were cultured overnight. The fluorescence intensity from SARS-CoV-2 infection was determined, and cell morphology was imaged at 24 h after infection. Diluted Listerine (3% *v*/*v*) reduced SARS-COV-2 infection by 40%, and CHG (1.5% *v*/*v*) reduced infection by 70%, without apparent impacts on cell morphology (Figure 3). Diluted Colgate Peroxyl (0.05% *v*/*v*) and povidone-iodine (0.1% *v*/*v*) appeared to have potent antiviral activities; however, disruption of cell morphology was apparent (Figure 3), indicating that the putative antiviral effect of these two agents was likely a consequence of cytotoxicity.

**Figure 4:**
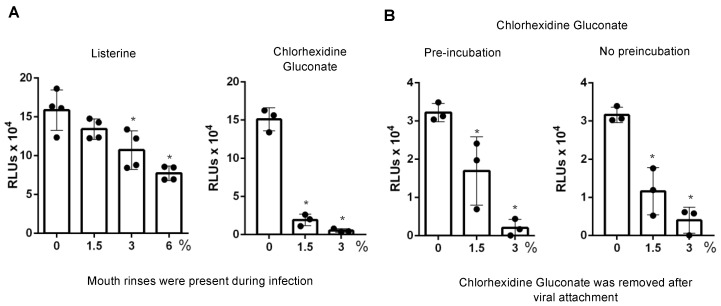
Effect of non-cytotoxic dilutions of Listerine and chlorhexidine gluconate on infection by pseudotyped SARS-CoV-2 viruses. (**A**) Pseudotyped SARS-CoV-2 virus (100 μL) was incubated with or without non-cytotoxic dilutions of Listerine or CHG at 37 °C for 30 min and then added to HeLa-hACE2 cells. After 1–2 h incubation, an additional 400 μL of Dulbecco’s Modified Eagle’s Medium (DMEM) 10% fetal bovine serum (FBS) was added to the cells without washing off the viruses or mouth rinses. Infected cells were cultured in the presence of mouth rinses for 2 days before measuring luciferase activity. (**B**) Pseudotyped SARS-CoV-2 viruses were pre-incubated with diluted CHG at 37 °C for 30 min (left panel) or without 30 min preincubation (right panel). Treated viruses were added to HeLa-hACE2 cells and incubated for 1 h at 37 °C. Cells were washed to remove residual mouth rinse and cultured for 2 days before measuring luciferase activity. The significance of differences between mouth rinse-treated viruses and mocked-treated controls (0%) was compared; * *p* < 0.05. Data are means ±SD.

We also used HIV pseudotyped luciferase virus particles expressing SARS-CoV-2 surface protein (spike, S) to assess the effect of non-cytotoxic diluted Listerine and CHG on viral infectivity. Unlike replication competent SARS-CoV-2 virus, which induces cytopathic effects after prolonged culture, HIV pseudotyped luciferase viruses provide a reliable, non-pathogenic vector for assessing viral infectivity. We confirmed that infection by pseudotyped SARS-CoV-2 was dependent on human hACE2, and that infection was neutralized by the anti-spike monoclonal antibody (Supplementary Figure S2). We determined the effect of diluted Listerine (1.5–6% *v*/*v*) and CHG (1.5% and 3% *v*/*v*), which had no or little effect on cell viability (Figure 2), on pseudotyped SARS-CoV-2 virus infection without washing off the mouth rinses during the infection. We found that 6% (*v*/*v*) Listerine had moderate anti-SARS-CoV-2 activity, whereas 1.5% or 3% (*v*/*v*) CHG suppressed viral infection by 88% and 97%, respectively (Figure 4A).

We also determined whether preincubation of viruses with CHG affected the degree of antiviral activity. For this, pseudotyped SARS-CoV-2 viruses were pretreated or not with CHG for 30 min at 37 °C before being added to target cells. In contrast to the experiment shown in Figure 4A, in which the mouth rinses were present during infection, here, the mixture of virus and CHG was removed, fresh medium was added, and cells were cultured for 2 days before measuring luciferase activity (Figure 4B). The effect of CHG with or without preincubation was comparable (Figure 4B). We found that the antiviral effect of 1.5% (*v*/*v*) CHG was less potent when CHG was present only during viral attachment (Figure 4B) compared with being continuously present during viral infection and incubation for 2 days (Figure 4A). The more pronounced antiviral activity of 1.5% (*v*/*v*) CHG in this experiment may be due to the effects of prolonged contact with CHG on the target cells, indicating the importance of minimizing mouth rinse-associated cytotoxicity in the infection assay.

### 3.3. Direct Effect of Mouth Rinses on Viruses

**Figure 5:**
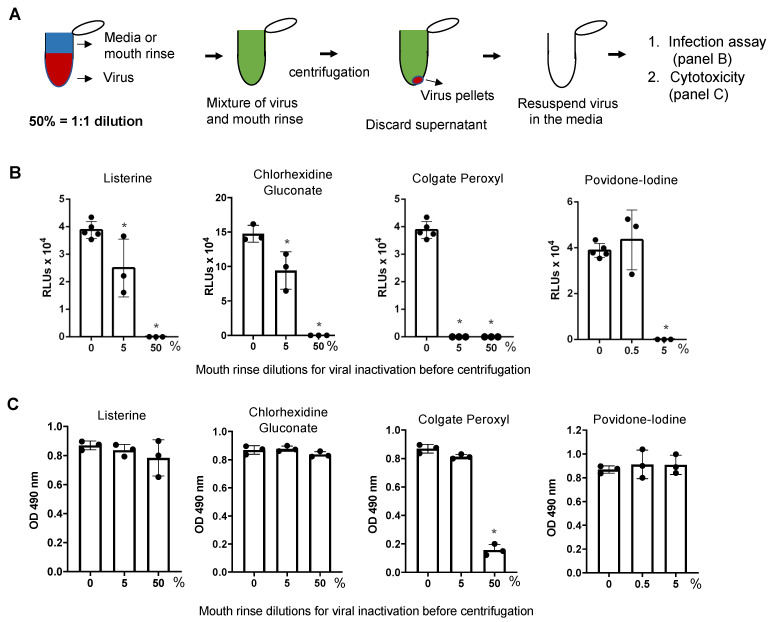
The effect of mouth rinses on SARS-CoV-2 viruses. (**A**) Experimental design. Pseudotyped luciferase reporter viruses expressing SARS-CoV-2 spike proteins were incubated with the indicated dilutions of mouth rinses at 37 °C for 30 min, followed by centrifugation and aspiration of the supernatant. Virus pellets were resuspended in the culture medium and added to HeLa-hACE2 cells for (**B**) infection assay and for (**C**) cytotoxicity assay as described in the Methods. Differences between mouth rinse-treated viruses and media controls (0%) were compared; * *p* < 0.05. Data are means ±SD and are representative of two independent experiments.

To assess the direct effects of the rinses on virus particles, pseudotyped SARS-CoV-2 viruses were incubated with mouth rinses for 30 min at 37 °C and were pelleted by centrifugation [24] prior to the infection assay. Note that centrifugation per se did not impact infectivity of the virus (data not shown). After removal of the supernatant containing the mouth rinse, viruses were resuspended in media and added to HeLa-hACE2 target cells (Figure 5A,B). We also assessed the effect of centrifugation and virus resuspension on infectivity and cell viability to monitor the potential cytotoxic effects of residual mouth rinse on the cells (Figure 5C). Viruses treated with 50% (*v*/*v*) Listerine, 50% (*v*/*v*) CHG, 50% (*v*/*v*) Colgate Peroxyl, or 5% (*v*/*v*) povidone-iodine completely lost infectivity (Figure 5B); the apparent antiviral effect of 50% (*v*/*v*) Colgate Peroxyl was associated with cell toxicity (Figure 5C). Treatment with 5% (*v*/*v*) Listerine or CHG had a moderate antiviral effect, whereas 5% (*v*/*v*) Colgate Peroxyl or 5% (*v*/*v*) povidone-iodine completely inactivated the viruses. All mouth washes at non-cytotoxic levels exhibited antiviral activity. Colgate Peroxyl and povidone-iodine had greater inhibitory effects on the viruses than CHG or Listerine.

Unlike high concentrations of Colgate Peroxyl and povidone-iodine, whose antiviral activities were associated with cytotoxicity, higher concentrations of Listerine and CHG exhibited potent antiviral effects without cytotoxicity. We asked whether preincubation of the virus with Listerine or CHG was required to achieve their direct effect on the virus.

Mouth rinses were added to the virus, mixed, and immediately centrifuged at 4 °C. Supernatants containing the mouth rinses were discarded. Viruses were then resuspended in media and added to target cells. The viral inhibition profiles of Listerine and CHG without preincubation (Supplementary Figure S3) were comparable to those with 30 min incubation (see Figure 5B).

**Figure 6:**
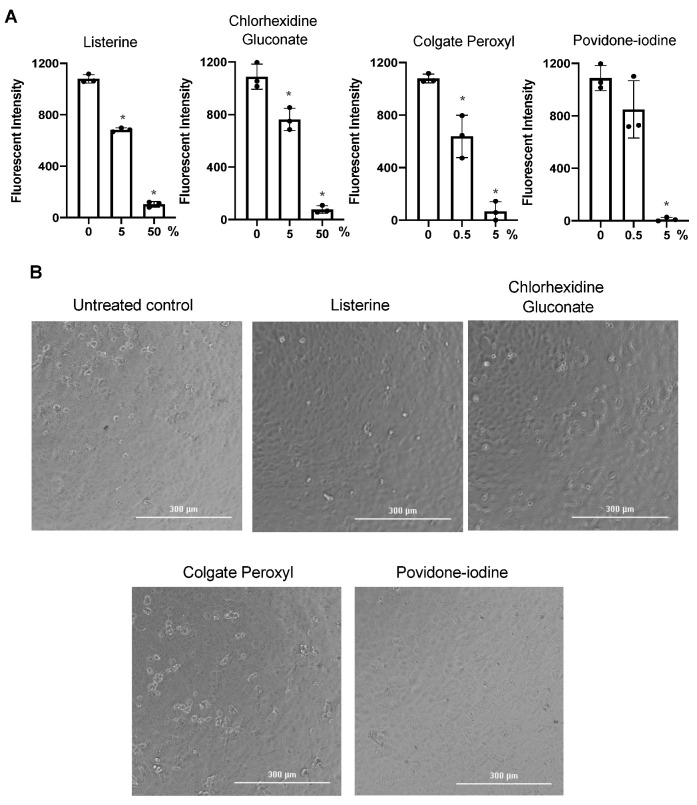
The effect of mouth rinses on replication-competent SARS-CoV-2 viruses. (**A**) Mouth rinses and povidone-iodine at the indicated dilutions were added to replication-competent SARS-CoV-2 expressing mNeonGreen (1 × 10^6^ plaque-forming units). Treated and untreated viruses were immediately pelleted by centrifugation, and the supernatant was aspirated. Virus pellets were resuspended in Fluorobrite medium with 2% FBS and added to Vero cells. Viral infection was determined by measuring fluorescence intensity at 24 h after infection. (**B**) Images of infected cells with or without mouth rinse treatment were acquired when the plate was read. Differences between mouth rinse- and medium control (0%)-treated viruses were compared; * *p* < 0.05. Data are means ±SD and are representative of two independent experiments.

We further confirmed the direct effect of mouth rinses on SARS-CoV-2 viral infectivity using replication-competent viruses expressing mNeonGreen. Note that there was no preincubation of viruses with mouth rinses. Similar to the results using pseudotyped viruses expressing spike proteins, 50% (*v*/*v*) Listerine, 50% (*v*/*v*) CHG, 5% (*v*/*v*) Colgate Peroxyl, and 5% (*v*/*v*) povidone-iodine significantly blocked viral infectivity; 0.5% (*v*/*v*) povidone-iodine was not active against the virus, whereas 0.5% (*v*/*v*) Colgate Peroxyl had moderate antiviral activity; 5% (*v*/*v*) Listerine and 5% (*v*/*v*) CHG had a moderate antiviral effect (Figure 6A,B). In contrast to infected cells with exposure to highly diluted Colgate Peroxyl and povidone-iodine during the infection, which was associated with cell death (Figure 3), there was no apparent cell death in cells infected by viruses after the removal of mouth rinses by centrifugation. Taken together, Listerine and CHG blocked SARS-CoV-2 viral infectivity with minimal cytotoxicity, whereas highly diluted Colgate Peroxyl and povidone-iodine significantly inactivated viruses but their antiviral effects were associated with severe cytotoxicity.

## 4. Discussion

Unlike SARS-CoV (SARS) and Middle East Respiratory Syndrome (MERS)-CoV, which caused thousands of cases and 700–800 deaths, SARS-CoV-2 appears to be more highly transmissible [25]. The SARS-CoV-2 virus is detectable in saliva from infected individuals without symptoms or with mild symptoms [26], suggesting that a strategy of suppressing the viral load in the oral cavity may reduce viral spread. Indeed, results from in silico and in vitro studies suggest the potential use of mouth rinses to reduce SARS-CoV-2 spread [15,16,17]. Previous studies were conducted to assess the antiseptic effect of povidone-iodine on several respiratory viruses, including SARS-CoV, MERS-CoV, and influenza A subtype H1N1, using virus-mediated cytopathic effects [27]; however, they did not exclude possible antiseptic-associated cytotoxicity. Similarly, published data on the effect of mouth rinses on SARS-CoV-2 infection did not distinguish the impact of mouth rinses on cell viability in efforts to determine the direct effects of mouth rinses on viral infection [18,19,20]. The apparent effective dosing of the antiseptic rinse on viral infectivity can be misleading when the putative antiviral effect is accompanied by a cytotoxic effect. Our experiments were designed to discriminate between the cytotoxic effects of the mouth rinses and the effects of the rinses on the infectivity of the virus. Indeed, we found that the antiviral effects of highly diluted Colgate Peroxyl and povidone-iodine were the consequence of cytotoxicity when the agents were present during a 24 h infection assay. We obtained similar results using both replication-competent viruses and pseudotyped SARS-CoV-2 viruses, indicating the reproducibility of the assays. Our results point to the need to distinguish between cytotoxicity and antiviral activity for in vitro analysis (e.g., by removing excess mouth rinse before infection). Our assays, in which the virus but not the infected cells were exposed to mouth rinses (Figure 5 and Figure 6), differed from previous studies [18,19,20], in which infected cells were exposed to mouth rinses and antiseptics for several days.

All mouth rinses tested (all products diluted 1:1 with culture medium, 50% *v*/*v*) had cytotoxic effects on cells. We found the cytotoxicity of Colgate Peroxyl > povidone-iodine > CHG > Listerine. Similar trends were observed in both HeLa-hACE2 and oral epithelial cells. Mouth rinse-induced cytotoxicity was more pronounced in cells with 2 h incubation than with 20 s incubation. When CHG was present during a 2-day infection period, 1.5 and 3% (*v*/*v*) CHG suppressed SARS-CoV-2 infection by nearly 99% (Figure 4). However, 1.5% (*v*/*v*) CHG was less potent when the mouth rinse was only present during viral attachment. Importantly, when assessing the effect of CHG on the viruses after removal of the mouth rinse during the infection, 5% (*v*/*v*) CHG had only a moderate effect, reducing infection by 35–55%. Similarly, the potent “antiviral” effects of 0.1% (*v*/*v*) povidone-iodine and 0.05% (*v*/*v*) Colgate Peroxyl that were observed when antiseptics were present during infection were found by cell image analysis to be due to antiseptic-associated cytotoxicity (Figure 3). In fact, we found that 0.5% (*v*/*v*) povidone-iodine had little effect on either replication competent or pseudotyped viruses if the povidone-iodine was removed from the virus before infection (Figure 5 and Figure 6). Our results show the importance of considering the potential cytotoxicity of putative antiviral agents when assessing their antiviral activities. Despite our finding that commercially available mouthwashes had some degree of cytotoxicity, these formulations are well tolerated in clinical use. The ability to determine the antiviral effects of these mouth washes independent of their cytotoxicity is important, however, for translating these laboratory results into clinical studies.

Both 5% (*v*/*v*) Colgate Peroxyl and 5% (*v*/*v*) povidone-iodine inactivated the virus effectively, whereas 50% Listerine and 50% CHG were required to inactivate the virus. We found that preincubation with mouth rinses was not required for their antiviral activities, indicating that the effect of mouth rinses on the virus occurs rapidly on contact. Determination of the antiviral activity of diluted mouthwash is important, since the salivary flow in the oral and pharyngeal cavities will dilute the activity following application. Thus, assessing the effects of highly dilute mouth rinses over time may help establish the frequency of rinsing necessary for optimal clinical benefits.

**Figure 7:**
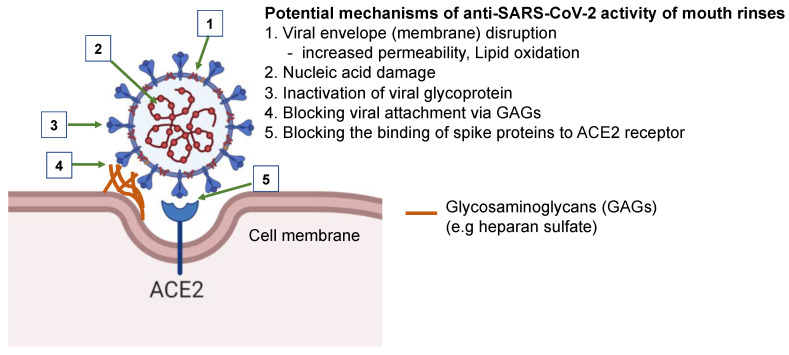
Potential mechanisms of the anti-SARS-CoV-2 activity of mouth rinses. Mouth rinses may inhibit SARS-CoV-2 infection by disruption of the viral envelope and viral nucleic acid, alteration of glycoprotein structure, blocking viral attachment to glycosaminoglycans on cell surface through changing charges on glycoproteins, and inhibiting the binding of SARS-CoV-2 spike proteins to angiotensin-converting enzyme 2 (ACE 2) receptors.

The differential antiviral and cytotoxicity profiles of these mouth rinses suggest that their antiviral mechanisms are not all the same. The underlying mechanism of the antiviral activity of mouth rinses and their active ingredients remains to be determined, although potential mechanisms by which mouth rinses inhibit SARS-CoV-2 have been the subject of speculation. In silico and in vitro studies have suggested the potential use of mouth rinses to reduce SARS-CoV-2 spread [15,16,17]. The active compounds in mouth rinses may block infection by altering/disrupting viral envelopes (membranes), nucleic acids, and viral proteins (Figure 7). For example, the key ingredient in Colgate Peroxyl is hydrogen peroxide, which is known to increase cell membrane permeability and cause DNA damage [28,29]. Hydrogen peroxide may inactivate viruses through lipid oxidation and/or nucleic acid damage. Povidone-iodine blocks influenza A virus by acting on the viral glycoproteins hemagglutinin and neuraminidase, resulting in the inhibition of the binding of virus to cells [30]. Chlorhexidine, a cationic molecule, reduces the infectivity of enveloped viruses, such as HIV and respiratory syncytial virus, and non-enveloped viruses, such as rotavirus and hepatitis A virus [31], suggesting that its action is not simply mediated through membrane disruption. Listerine, an essential oil-based mouth rinse, has been shown to inhibit infection by HIV and herpes simplex virus 1 [32], and may reduce the infectivity of viruses through altering the hydrophobicity of the viral glycoproteins necessary for viral attachment.

In conclusion, all mouth rinses tested inactivated replication-competent SARS-CoV-2 viruses and pseudotyped viruses expressing spike proteins. The cytotoxic effects of mouth rinses should be considered when assessing their antiviral activities. Since diluted Listerine and CHG exhibited no cytotoxic effects, these products may be good candidates to reduce virus spread. Studies of the antiviral effects of mouth rinses are needed for determining their clinical efficacy in reducing virus spread, particularly in asymptomatic individuals.

## Figures and Tables

**Table 1:** Mouth rinse and antiseptic products used in this study.

Products	Manufacturers	Active Ingredients
Listerine Antiseptic Original	Johnson & Johnson Consumer Inc., Skillman, NJ, USA	20–30% ethanol Thymol 0.064% Methyl salicylate 0.06% Menthol (racementhol) 0.042% Eucalyptol 0.092%
Chlorhexidine gluconate	Xttrium Laboratories Inc., Mount Prospect, IL, USA	0.12% chlorhexidine
Colgate Peroxyl	Colgate-Palmolive Inc., New York, NY, USA	1.5% *w*/*v* hydrogen peroxide
Povidone-iodine	CVS Pharmacy Inc., Woonsocket, RI, USA	10% solution (1% available iodine)

## Data Availability

All results generated for this study have been included in the current manuscript.

## Supplementary Materials

The following are available online at https://www.mdpi.com/2076-0817/10/3/272/s1. Figure S1: A workflow diagram of infection assays used in the study, Figure S2: HIV pseudotyped virus expressing SARS-CoV-2 spike proteins, Figure S3: Preincubation of viruses was not required for the inhibitory effect of mouth rinses.
