# Supplementary material for: Differential Effects of Antiseptic Mouth Rinses on SARS-CoV-2 Infectivity In Vitro
Source: Pathogens. 2021 Mar 1;10(3):272. doi: 10.3390/pathogens10030272 (PMC8001756; doi:10.3390/pathogens10030272)
Supplement: Supplementary file 1 [file pathogens-10-00272-s001.pdf]

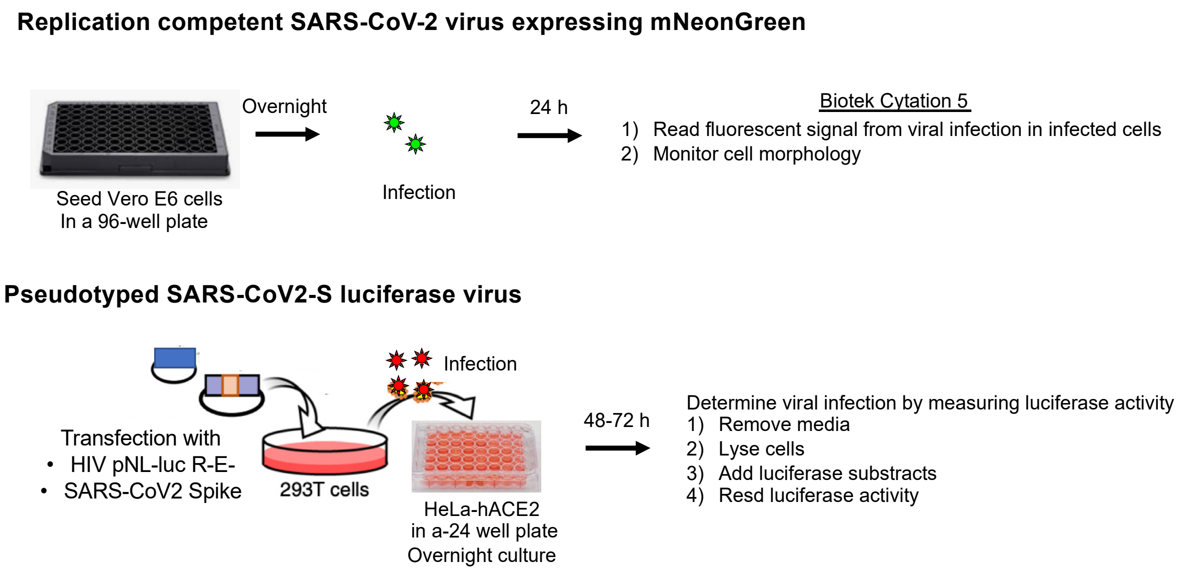


**Figure S1. A workflow diagram of infection assays used in the study.**

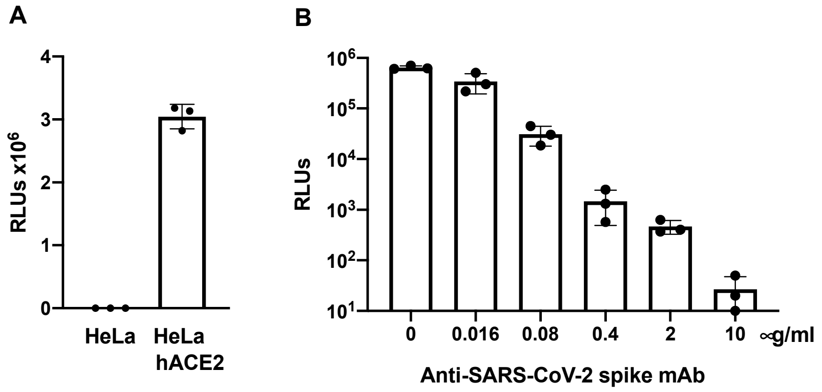


**Figure S2. HIV pseudotyped virus expressing SARS-CoV-2 spike proteins** (A) Parental HeLa cells or HeLa cells overexpressing hACE2 were infected with HIV pseudotyped SARS-CoV-2 (~20 ng p24 per well; 48-well plate) for 1 h. Cells were washed, and then cultured for 3 days before measurement of luciferase activity in infected cells. The results show that the infection by pseudotyped luciferase virus expressing SARS-CoV2 spike proteins was hACE2-dependent. (B) Pseudotyped SARS-CoV-2 virus was incubated with different concentrations of monoclonal antibody against SARS-CoV-2 spike proteins for 1 h before infection of HeLa-hACE2 cells. Luciferase activity was measured at 48 h post-infection. The result confirmed that anti-spike protein antibody blocked infection by pseudotyped virus indicating that infection was mediated by the spike proteins.

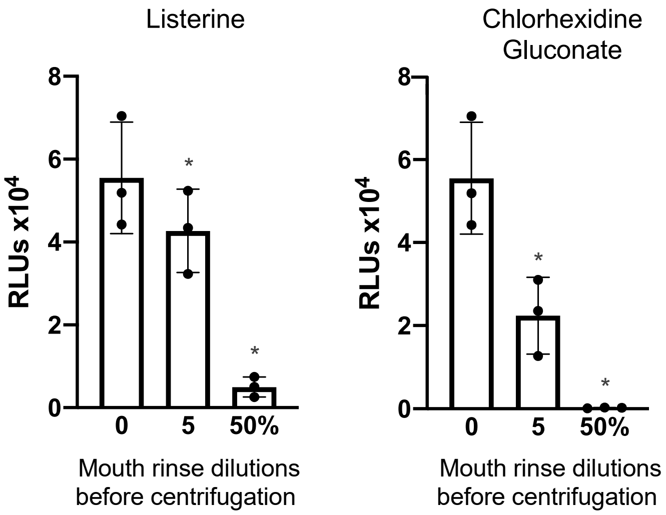


**Figure S3. Preincubation of viruses was not required for the inhibitory effect of mouth rinses.** Mouth rinses were added to pseudotyped SARS-CoV-2 viruses, mixed, and immediately centrifuged. Supernatants were aspirated, virus pellets were resuspended in medium, and added to HeLa-hACE2 cells. Infection was determined by measuring luciferase activity on post-infection day 2. Data are mean ±SD. Differences between mouth rinse-treated viruses and medium control (0%) were compared; * $p < 0.05$. The anti-viral profiles of Listerine and CHG were comparable to the results with pre-incubation of viruses with mouth rinses for 30 min shown in Fig 5, indicating that pre-incubation of viruses with mouth rinses was not required to inactivate the viruses.
